# Mental Treatment for Crime

**Published:** 1920-12-11

**Authors:** Charles W. J. Tennant

**Affiliations:** Committee on Publication. Christian Science Committees on Publication, Talbot House, Arundel Street, Strand, W.C. 2


*To the Editor of* The Hospital.

### Mental Treatment for Crime.

*To the Editor of* The Hospital.

Sir,—An article entitled "Mental Treatment for Crime," appearing in your issue of the 20th inst., contains a reference to Christian Science which might leave a wrong impression in the minds of your readers as to its teaching and practice.

Christian Science mind healing differs from all other so-called psycho-therapeutic methods in that it utilises the Divine energies of spiritual truth, revealed through obtaining the mind of Christ. It draws a clear line of distinction between the Divine mind and the carnal or human mind. The point of view of the former is spiritual, immortal, and eternal; that of the latter is material, mortal, and temporal. In the carnal mind, which St. Paul terms "enmity against God," originate the beliefs of sin, disease, and death. This so-called mind knows nothing of God or

**The Editor's Letter Box—(*continued*).**

the spiritual universe; it has no remedy for its own mistakes, for all its beliefs are of the nature of "temporal falsities." The Divine mind, on the other hand, is the mind that was in Christ Jesus. It enabled him to comprehend all things from a spiritual view-point, which is God's view-point, thus giving him power "to subdue material conditions" ("Unity of God," by Mary Baker Eddy, p. 42).

Christian Science treatment consists in replacing the false beliefs of the carnal or mortal mind by the spiritual truths of the Divine mind. This is what St. Paul meant when he said, "Be ye transformed by the renewing of your mind." The declaration of Christ Jesus, "Ye shall know the truth, and the truth shall make you free," has precisely the same meaning. Christian Science heals morally as well as physically, and in this it differs from all the methods in which the carnal or human mind employs suggestion, mesmerism, or hypnotism.—Yours faithfully,

Charles W. J. Tennant,
Committee on Publication.

Christian Science Committees on Publication,
Talbot House, Arundel Street, Strand, W.C. 2,
November 26, 1920.

